# Associations of Childhood Adversity and Polygenic Scores with Substance Use Initiation and Disorder Severity – ERRATUM

**DOI:** 10.1017/S0033291725100615

**Published:** 2025-05-22

**Authors:** Jackson F. SooHoo, Christal N. Davis, Angela Han, Zeal Jinwala, Joel Gelernter, Richard Feinn, Henry R. Kranzler

**Affiliations:** 1 Center for Studies of Addiction, Department of Psychiatry, University of Pennsylvania School of Medicine, Philadelphia, PA, USA; 2 Mental Illness Research, Education, and Clinical Center, Crescenz VAMC, Philadelphia, PA, USA; 3 Department of Psychiatry, Yale University School of Medicine, New Haven, CT, USA; 4 VA Connecticut Healthcare System, West Haven, CT, USA; 5 Department of Medical Sciences, Frank H. Netter School of Medicine, Quinnipiac University, North Haven, CT, USA

When this article was originally published in Psychological Medicine it contained an error in its title. This has now been updated

The authors apologise for this error.
